# An investigation of the psychological stress of medical staff in Shanghai shelter hospital during COVID-19

**DOI:** 10.3389/fpsyg.2023.1083793

**Published:** 2023-03-09

**Authors:** Yaqing Zhou, Aiming Liu, Zunguo Pu, Minlu Zhou, Hongsheng Ding, Jia Zhou

**Affiliations:** ^1^Department of Critical Care Medicine, Affiliated Hai'an Hospital of Nantong University, Nantong, Jiangsu, China; ^2^Department of General Surgery, Affiliated Hai'an Hospital of Nantong University, Nantong, Jiangsu, China; ^3^Department of Psychiatry, Affiliated Nantong Fourth People's Hospital, Nantong, Jiangsu, China

**Keywords:** COVID-19, medical workers, mental health, stress, intervention

## Abstract

**Objective:**

The purpose of this study was to assess the psychological status of medical workers from other locations who helped support the fight against COVID-19 in Shanghai and to provide a basis for psychological crisis intervention plans under designated emergencies.

**Methods:**

While supporting the Shanghai Lingang Shelter Hospital, we investigated 1,097 medical staff from other cities working in the hospital. A questionnaire comprising the general information questionnaire, health questionnaire depression scale, generalized anxiety scale, insomnia severity index scale, and mental health self-assessment questionnaire was used.

**Results:**

There were no statistically significant differences in the incidence rates of anxiety, depression, and sleep disorders among subjects of different genders, ages, and educational levels. There were statistically significant differences in the incidences of anxiety, depression, stress response, and sleep disturbance among subjects with different levels of worry about COVID-19.

**Conclusion:**

During the COVID-19 pandemic, the Lingang Shelter Hospital team experienced more psychological pressure, suggesting that medical institutions should pay attention to the mental health of frontline medical workers during COVID-19 and prepare psychological intervention measures for team members.

## 1. Introduction

Health refers to a person’s physical, mental, and social well-being. The World Health Organization (WHO) proposes that health is not only the absence of disease in the body, but also includes good mental health and social adaptation (Fitzpatrick, 2010). According to this definition of health, mental health is an important component of health (Smith, 2014). However, in people’s daily lives and work, mental health is often overlooked, especially for medical workers. With a good psychological state in work and occupation, you are able to give full play to one’s abilities and living an efficient life (Ohrnberger et al., 2017).

Public health emergencies refer to major infectious epidemics, mass unexplained diseases, major food and occupational poisonings, and other events that seriously affect public health, and that occur suddenly and may cause serious damage to public health (Khan et al., 2018).

COVID-19 is an acute respiratory disease (Li et al., 2021a) which has affected people globally. At the onset of symptoms, patients develop fever, fatigue, a dry cough, and upper respiratory tract symptoms, such as nasal congestion and a runny nose (Ochani et al., 2021). As the disease progresses, patients are prone to dyspnea, acute respiratory distress syndrome, septic shock, metabolic acidosis, and coagulation dysfunction, and severe cases directly endanger their lives (Majumder and Minko, 2021). With variations of the virus, Omicron spreads faster, is more infectious, and is more widely spread than the earlier COVID-19 variants (Ren et al., 2022; Tian et al., 2022). Not only does this cause physical and mental distress at an individual level, but psychological stress in other groups can also lead to a variety of adverse factors (Killgore et al., 2020; Wang et al., 2020). In particular, frontline medical staff are required to be in close contact with patients with COVID-19 (Buysse, 2014; Sims et al., 2022) in order to treat them.

The psychological crisis intervention of the COVID-19 pandemic is not only aimed at confirmed patients, suspected patients, and quarantined personnel, but all medical staff, and some social organizations and groups also have a need for mental health intervention (Xiang et al., 2020) because they are also at risk of infection. They shoulder the responsibility for treating diseases and promoting patient recovery. During the pandemic, they faced special nursing environments and methods, and needed to wear professional protective equipment (PPE) in an environment fully exposed to the COVID-19 virus, which may have caused high levels of psychological stress, leading to sleep disturbance, anxiety, depression, and other negative emotions (Chen et al., 2005; Killgore et al., 2020). This study aimed to understand the psychological state and characteristics of frontline medical workers during the COVID-19 outbreak and to provide a basis for specifying psychological crisis intervention plans under major public safety events.

## 2. Related work

Depression is a mood disorder caused by the interaction of multiple factors, with clinical manifestations such as low mood, loss of interest, and corresponding cognitive and behavioral changes (Bonde, 2008). In severe cases, psychotic symptoms, such as hallucinations and delusions, may occur (Rakel, 1999). Depression tends to recur and repeated episodes tend to result in poor outcomes (Amore et al., 1995).

Globally, depression causes patients to lose more years of life than any other condition (Gherardi-Donato et al., 2015; Saxena et al., 2015). However, due to a lack of formal and effective treatment methods and mental health resources, most patients with depression are not effectively diagnosed and treated (Saxena et al., 2015). Anxiety is a kind of irritable mood caused by various factors, which contains the manifestations of sadness, tension, and fear, and is accompanied by symptoms of autonomic nervous dysfunction such as palpitations and frequent urination (Craske and Stein, 2016).

Depression and anxiety are common mood and neurotic disorders (Lee et al., 2016). Medical caregivers work in the clinical frontline for extended periods of time. Owing to the high workload, heavy tasks, and long working hours, an increasing number of caregivers feel frustrated and anxious at work (Liu Q. et al., 2020). Mental health problems associated with depression have received widespread attention (McLeod et al., 2016). Medical workers are in a tense work environment, and there is increased occupational pressure (Tan et al., 2020). The main sources of stress are work and family pressures (Wu et al., 2010). Medical workers are at an increased risk of psychological disorders, such as burnout syndrome and post-traumatic stress disorder, due to their stressful work environment. Among them, depression and anxiety have become some of the most prominent psychological problems (Michie and Williams, 2003; Cares et al., 2015; Johnson et al., 2017). Many studies have shown that medical workers have a higher incidence of depression and anxiety than the general population (Galbraith et al., 2014; Hernandez et al., 2016). A study at a hospital in Iran found that 45.4% of medical workers had mental disorders. The prevalence of anxiety and somatic symptoms was 43.2%, and that of other types of mental disorders was 34.5%. The prevalence rates of depression and social dysfunction were 11.2 and 79.5%, respectively. Moreover, shift work is significantly associated with anxiety, and marital status is significantly associated with depression and social dysfunction (Andrade et al., 2003). A study in South Korea showed that after adjusting for sociodemographic and health behavioral factors, medical workers who worked shifts had 1.519 times greater severity of depressive symptoms than those who worked regular shifts, and that among nurses working shifts, depression was 1.519 times higher. The prevalence is high (Lee et al., 2016). Roskoden et al. (2017) found that the mental health problems of medical workers with heavy workloads are mainly manifested in three aspects: nervousness, insomnia, anxiety and irritability, and low self-esteem. A 2017 study showed that the current state of occupational stress among healthcare workers cannot be ignored. The factors influencing occupational stress include work, individual, and psychological factors related to individuals (Clough et al., 2017). A study on depression and related factors among psychiatric nurses in Taiwan found that 12.8% of nurses had mild depression, 7.1% had moderate depression, and 7.8% had severe depression (Lin et al., 2010). These studies have shown that the psychological problems of medical workers are very serious.

Based on the above, it is obvious that the psychological status of medical workers is problematic. In addition, it has been exacerbated by COVID-19 which has increased their workload and this has gained the attention of the medical community. In addition, with the development and mutation of the virus, the Omicron strain spread faster and was more contagious than the original strain. This increased the risk of infection of frontline healthcare workers (Gómez-Ochoa et al., 2021). COVID-19 has made the original working environment more complicated and has aggravate the original psychological problems causing more pressure. At the height of COVID-19, medical workers who were quarantined, worked in COVID-19 Specialized Hospital, or had family or friends infected with COVID-19, had considerably more anxiety, depression, frustration, fear, and post-traumatic stress than others (Xiang et al., 2020). Liu S. et al. (2020) found that COVID-19 increased the negative emotions and physical symptoms of medical staff, leading to serious physical and mental discomfort. Studies also demonstrated that frontline medical workers suffered severe insomnia compared to non-frontline medical workers (Li et al., 2021b).

## 3. Research participants and methods

### 3.1. Participants

From April to May 2022, the participants included 1,097 medical staff from Shanghai Lingang Shelter Hospital, who were from Jiangsu Province’s medical team supporting Shanghai. The survey was registered with the China Clinical Trial Center with registration number ChiCTR1800020214.

### 3.2. Methods

An online questionnaire survey method was used using QR codes. The specific contents of the questionnaire were as follows:

1. General information such as sex, age, marital status, education level, exposure to COVID-19, concerns about COVID-19, and current health status.
2. The 9-item Patient Health Questionnaire (PHQ-9). The scale consists of nine items where each item is scored from 0 to 3 points, with the total score ranging from 0 to 27 points. A score of 0 means not at all, and a score of 3 means it occurs almost every day. Scoring criteria: 0–4 points for no depression; 5–9 points for mild depression; 10–14 points for moderate depression, and >15 points for severe depression. The effectiveness of the PHQ-9 in college students has been previously demonstrated (Levis et al., 2020).
3. Generalized Anxiety Disorder Scale (GAD-7). The scale consists of seven items, where each item is scored from 0–3, with the total score ranging from 0–21. A score of 0 means not at all, and a score of 3 means that it occurs almost every day. The scoring criteria: 0–4 points for no anxiety; 5–9 points for mild anxiety; 10–14 points for moderate anxiety, and >15 points for severe anxiety.
4. Insomnia Severity Index (ISI). A total of seven items were included, with a total score of 28 points, which was used to evaluate the severity of insomnia in the last month. Scoring criteria: 0–7 points, no insomnia; 8–14 points, subclinical insomnia; 15–21 points, moderate insomnia; 22–28 points, severe insomnia.
5. Mental Health Self-Assessment Questionnaire (Self-reporting questionnaires 20, SRQ-20). The scale has 20 items, and each item is scored on a two-point scale. Scoring standard: Nine points are the cut-off value, and less than eight points indicate no obvious stress response. A score greater than eight indicates a stress response that requires attention. The SRQ-20 guidebook published by the WHO comprehensively analyzes its validity and has a good predictive ability.

### 3.3. Statistical analysis

SPSS software (Version 23.0) was used for data analysis. Measurement data and count data are expressed as mean ± standard deviation and percentage, respectively.

## 4. Results

The number of valid participants in the questionnaire survey was 1,097, and the response rate was 100%. Participants included 19 males and 1,078 females. The age distribution ranged from 18 to 60 years, with an average age of 29.00 ± 5.88 years old. Details are presented in Table 1.

**Table 1:** Basic information of the medical workers included in the study.

Basic information		Number of cases	Percentage (%)
Age	18–25	326	29.72
	26–45	742	67.64
	46–60	29	2.64
Marital status	Married	514	46.86
	Unmarried	574	52.32
	Divorced	9	0.82
Education level	Phd	1	0.09
	Master	7	0.64
	Bachelor	617	56.24
	Associate degree	472	43.03
Contact with COVID-19	Family members or relatives infected COVID-19	1	0.09
	A coworker or neighbor contracted COVID-19	36	3.28
	No history of exposure to COVID-19	1,059	96.54
	COVID-19 patients	1	0.09
Concerns about COVID-19	Do not worry	75	6.84
	A little worried	505	46.03
	More worried	111	10.12
	Worried	227	20.69
	Very worried	179	16.32
Current health status	Good	1,008	91.89
	Not good	89	8.11

Among medical workers who participated in the survey, 10.12% experienced stress reactions. The incidence of depressive symptoms was high, with moderate and severe depression accounting for 9.66% of the total. The proportion of people with moderate and severe anxiety was 3.46% and the proportion of people with moderate and severe insomnia was 2.83%. Details are presented in Table 2.

**Table 2:** Overall anxiety, depression, stress response and insomnia.

Item	Degree grading	Number of cases (NC)	Percentage (%)
PHQ-9	None	689	62.81
	Mild	302	27.53
	Moderate	83	7.57
	Severe	23	2.10
GAD-7	None	795	72.47
	Mild	264	24.07
	Moderate	33	3.01
	Severe	5	0.46
ISI	None	863	78.67
	Mild	203	18.51
	Moderate	27	2.46
	Severe	4	0.36
SRQ	No stress response	986	89.88
	Have a stress response	111	10.12

The results of the adjustment of depression in 1,097 medical workers during the previous 2 weeks (from the date of answering the questionnaire) showed that the highest incidence of depression-related manifestations was sleep disturbance, loss of pleasure, and lack of energy. Among them, the proportions of the above symptoms that existed more than half of the time in the previous 2 weeks reached 12.03, 10.48, and 10.12%, respectively. Details are presented in Table 3.

**Table 3:** Items of the PHQ-9 scale.

Item	No	A few days	more than half	almost everyday
	NC (%)	NC (%)	NC (%)	NC (%)
Restless	498 (45.40)	542 (49.41)	48 (4.38)	9 (0.82)
Uncontrollable worry	666 (60.71)	379 (34.55)	46 (4.19)	6 (0.55)
Worry about various things	537 (48.95)	491 (44.76)	55 (5.01)	14 (1.28)
Tension cannot relax	758 (69.10)	309 (28.17)	22 (2.01)	8 (0.73)
Fidgeting	914 (83.32)	170 (15.50)	12 (1.09)	1 (0.09)
Easily disturbed and excited	687 (62.63)	363 (33.09)	38 (3.46)	9 (0.82)
Worry about something bad happening	778 (70.92)	294 (26.80)	21 (1.91)	4 (0.36)

Among anxiety-related manifestations, the highest proportions are worrying about various things, restlessness, and uncontrollable worry. Among them, the proportions of the above symptoms that existed more than half of the time in the previous 2 weeks were 6.29, 5.20, and 4.74%, respectively. Details are presented in Table 4.

**Table 4:** Items of GAD-7 Scale.

Item	No	A few days	more than half	almost everyday
	NC (%)	NC (%)	NC (%)	NC (%)
Loss of pleasure	481 (43.85)	501 (45.67)	88 (8.02)	27 (2.46)
Depressed mood	666 (60.71)	374 (34.09)	46 (4.19)	11 (1.00)
Sleep disorder	602 (54.88)	363 (33.09)	95 (8.66)	37 (3.37)
Lack of energy	534 (48.68)	452 (41.20)	75 (6.84)	36 (3.28)
Eating disorder	647 (58.98)	356 (32.45)	73 (6.65)	21 (1.91)
Low self-esteem	845 (77.03)	215 (19.60)	29 (2.64)	8 (0.73)
Difficulty concentrating	674 (61.44)	303 (27.62)	87 (7.93)	33 (3.01)
Anxious	895 (81.59)	170 (15.50)	27 (2.46)	5 (0.46)
Negative idea	1,009 (91.98)	76 (6.93)	8 (0.73)	4 (0.36)

Among the depression-related manifestations, the highest incidences were fatigue, difficulty in making decisions, feeling afraid, nervousness, worry, and poor sleep. The details are presented in Table 5.

**Table 5:** Items of the SRQ-20 Scale.

Item	SC	Probability of occurrence (%)
Frequent headaches	166	15.13
Poor appetite	64	5.83
Poor sleep	285	25.98
Easily startled	203	18.51
Trembling	80	7.29
Feeling restless, nervous, worried	302	27.53
Indigestion	142	12.94
Unclear thinking	91	8.30
Not feeling well	201	18.32
Cry more than before	150	13.67
Hard to have fun	194	17.68
Difficult to make decisions	358	32.63
Daily work is painful	95	8.66
Not being hungry can make a difference in life	97	8.84
Loss of interest in things	82	7.47
Feel worthless	69	6.29
Want to end life	76	6.93
Feel tired	133	12.12
Upset stomach	166	15.13
Get tired easily	433	39.47

Satisfaction with sleep was the lowest (26.16%), followed by difficulty falling asleep (42.94%), and the impact of sleep on daily functions (41.39%). Among them, 32.18, 12.76, and 9.30% of the above symptoms had moderate or greater severity in the previous 2 weeks, as shown in Table 6.

**Table 6:** Items of the ISI scale (*n* = 1,097).

Item	No	Mild	Moderate	Severe	Very severe
	NC (%)	NC (%)	NC (%)	NC (%)	NC (%)
Difficulty falling asleep	626 (57.06)	331 (30.17)	102 (9.30)	32 (2.92)	6 (0.55)
Difficulty maintaining sleep	712 (64.90)	270 (24.61)	95 (8.66)	15 (1.37)	5 (0.46)
Wake up early	692 (63.08)	294 (26.80)	83 (7.57)	26 (2.37)	2 (0.18)
Satisfaction with sleep	287 (26.16)	457 (41.66)	248 (22.61)	80 (7.29)	25 (2.28)
Affect daily functioning	643 (58.61)	352 (32.09)	88 (8.02)	12 (1.09)	2 (0.18)
Affect quality of life	756 (68.92)	272 (24.79)	55 (5.01)	12 (1.09)	2 (0.18)
Level of worry/pain	706 (64.36)	291 (26.53)	83 (7.57)	15 (1.37)	2 (0.18)

## 5. Discussion

Public health emergencies have become the focus of social attention in recent years, such as the “atypical pneumonia” in 2003 and the outbreak of Influenza A (H1N1) in 2009, which directly posed serious harm to public health. Medical workers directly respond to and handle public health emergencies, and studies have shown that in the event of public health emergencies, the emotional changes of medical staff are a process from stress with destructive or unpleasant experiences to excessive stress with constructive or exciting experiences. This change trend is of positive significance for alleviating or reducing the stress response. Two months after the outbreak of severe acute respiratory syndrome (SARS), a survey study on the psychological status of medical staff showed that under public health emergencies, the negative emotions of medical staff were widespread (Salari et al., 2020) and others studied the mental health status of nurses during the sudden SARS outbreak and found that there were significant differences in the depression and anxiety of nurses in different occupational environments. A retrospective study on the psychological state of medical staff during the outbreak of Influenza A by Goulia et al. (2010) and others found that the psychological abnormalities of high-risk medical staff were mainly mood disorders and sleep disorders, and the three symptoms with the highest abnormal incidence were anxiety (41.46%), depression (30.32%), and sleep (23.27%). Studies have found that caregivers at moderate risk have lower levels of mental health than caregivers at high and low risk, and are more likely to show stress and lower coping skills. When an individual faces a crisis, there are a series of psychosomatic reactions, which mainly manifest as physical, emotional, cognitive, and behavioral. Simultaneously, the crisis is self-limiting; generally, the acute phase is 6 weeks, and the result may be well-adapted or maladaptive, and the individual’s response to the crisis varies greatly, which is related to the individual’s cognition, interpretation, and social support of the event. The front-line medical workers need to be in close contact with a variety of patients clinically, face the risk of possible infection, and may also face pressures such as inexperience and incomprehension. In the event of an epidemic, special protection will lead to physical discomfort and inconvenience at work, work fatigue, and worry about family members, and a good psychological state is the premise to better help others.

Front-line medical staff supported by external resources were at the center of pathogen exposure and were very susceptible to virus infection, which could have placed a strain on psychological endurance. At the same time, they stayed away from their loved ones for long periods of time, leading to observable depressive symptoms. The possibility of medical personnel becoming infected might negatively impact the confidence of the general public, and factors such as the increasing number of confirmed diagnoses and deaths, and enforced isolation might lead to decreased mental health (Chen et al., 2022). Many clinical staff have been moved into new roles and may be managing acutely unwell patients using unfamiliar equipment. Stress caused by high patient mortality rates, shortstaffing, concerns about infecting oneself or family members, and changing guidance on PPE can add to work pressure. This has raised concerns regarding the potential impact on the mental health of pandemic responders (Chen et al., 2020; Greenberg et al., 2020). In addition, since COVID-19 is highly contagious, they worry that their PPE is not guaranteed (Juan et al., 2020).

This study highlights that under the new crown pneumonia epidemic, 10.12% of medical workers experienced stress reactions, mainly manifested as being easily fatigued, struggling to make decisions, poor sleep, easily frightened, and feeling unhappy. The stress response is related to the degree of worry about the new crown pneumonia epidemic. Among those who were very worried about the epidemic, 21.79% had a stress response. At the same time, 24.07% had mild anxiety, and 3.46% had moderate and severe anxiety, mainly manifested as worrying about various things, restlessness, and uncontrollable worry. During the epidemic, keep abreast of the development of the epidemic, master the latest protective measures, and appropriately train medical workers. Implementing professional knowledge training and joint psychological intervention programs for medical staff in public health emergencies can promote professional operation skills, improve coping and first aid capabilities, and enhance psychological resilience, which may help to improve psychological stress. Moderate anxiety is also a protective factor for mental health, which can remind people to increase their alertness and enhance their awareness of protection.

The results of this study show that under the new crown pneumonia epidemic, some medical workers have depression, 27.53% of them have mild depression, and 9.66% of them have moderate and severe depression, mainly manifested as sleep disturbance, loss of pleasure and difficulty in concentrating. This result may be due to the fact that they were still active on the front-line, and their tense working state made them temporarily inattentive to changes in their physical and psychological conditions and even prompted a psychological defense, and the fatigue caused by adverse psychological effects and mental symptoms may appear later (Chen et al., 2022). This finding is consistent with the conclusions of previous studies. Nickell et al. (2004) and other studies on the impact of the SARS outbreak on social psychology showed that ~20% of the people had depression, and the incidence rate of medical workers was 45%. During the COVID-19 pandemic, sleep disorders, difficulty falling asleep, difficulty maintaining sleep, and low satisfaction with sleep were primary complaints (Bhat and Chokroverty, 2022). Studies have shown that, due to COVID-19, negative emotions of medical workers are closely related to the stress of destructive or unpleasant experiences, and the purpose of stress management is to control various emotional reactions in the best state (Sun et al., 2020). Targeting the psychological needs of medical workers in emergencies, such as caring about life and family, etc., can ease their worries and reduce psychological pressure (Evanoff et al., 2020). Under the active control of the state, the pandemic is preventable and controllable. In the early stage of the outbreak, mild emotional symptoms and physical reactions were within the normal range, and with the control of the pandemic, the state of medical workers will improve. Since the outbreak of Ebola virus and Zika virus, the WHO has begun to focus on multidisciplinary and comprehensive interventions to maintain the physical and mental health of infected people, non-infected people, people involved in rescue, media personnel, and administrative personnel, and improve general health as much as possible. On the current basis, according to the “Guiding Principles for Emergency Psychological Crisis Intervention in the Pneumonia Epidemic of COVID-19” issued by the National Health and Medical Commission, it is necessary to further investigate and analyze the psychological state of the relevant personnel during the pandemic, and gradually establish a specific psychological crisis intervention. Simultaneously, it is necessary to acknowledge the possible stress and emotional reactions of medical workers under the outbreak of the pandemic, follow changes to psychological states under stress response, and actively respond.

Through this study, we found that when public health events occur, frontline medical workers will experience greater psychological pressure than usual, which provides a basis for formulating psychological crisis intervention plans for medical workers when public health events occur.

The focus of this study is mainly on two aspects. First, research that affects the mental health of medical workers needs to further expand the sample size to provide a theoretical basis for improving and intervening in the psychological status of front-line medical workers. Second, in the future, frontline medical workers with mental health problems should be followed-up on, and targeted help and support should be provided.

## Data availability statement

The raw data supporting the conclusions of this article will be made available by the authors, without undue reservation.

## Ethics statement

The studies involving human participants were reviewed and approved by Medical Ethics Committee of Hai’an People’s Hospital is subordinate to Hai’an People’s Hospital. The patients/participants provided their written informed consent to participate in this study.

## Author contributions

All authors listed have made a substantial, direct, and intellectual contribution to the work and approved it for publication.

## Funding

The research is supported by Fund of Hai’an Hospital Affiliated to Nantong University (202204); Fund of Science and Technology Bureau of Nantong City, Jiangsu Province (JCZ2022122).

## Conflict of interest

The authors declare that the research was conducted in the absence of any commercial or financial relationships that could be construed as a potential conflict of interest.

## Publisher’s note

All claims expressed in this article are solely those of the authors and do not necessarily represent those of their affiliated organizations, or those of the publisher, the editors and the reviewers. Any product that may be evaluated in this article, or claim that may be made by its manufacturer, is not guaranteed or endorsed by the publisher.
